# Prospective comparative study on postoperative pain after single-visit root canal treatment using rotary and reciprocating kinematics

**DOI:** 10.1007/s10266-026-01316-4

**Published:** 2026-01-27

**Authors:** Muhammet Emin Çağlan, Fatma Pertek Hatipoğlu, Ömer Hatipoğlu

**Affiliations:** 1 Department of Endodontics, Recep Tayyip Erdoğan University, Rize, Türkiye; 2 Department of Restorative Dentistry, Recep Tayyip Erdoğan University, Rize, Türkiye

**Keywords:** Visual analog scale, Root canal treatment, Postoperative pain

## Abstract

This prospective clinical study aimed to compare postoperative pain and analgesic intake following single-visit root canal treatment using two rotary file systems with different kinematic motions: reciprocating (T-endo MUST, TeM) and continuous rotary (Scope RS Narrow, SRN). A total of 121 patients with vital mandibular premolars were prospectively allocated using an alternate, non-randomized approach to single-visit root canal treatment performed by the same operator. The primary outcome was postoperative pain intensity, measured using a 100-mm Visual Analog Scale (VAS) on day 3 after treatment. The secondary outcomes included the VAS scores recorded at 1, 5, and 7 days and the analgesic consumption. A responder analysis was conducted, defining responders as patients who achieved ≥ 30% or ≥ 50% reduction in pain or a VAS score < 10. Pain intensity decreased significantly over time in both groups (*p* < 0.001). The TeM group was associated with higher pain scores on the third postoperative day (8.31; 95% CI: 3.89–12.72) than the SRN group (3.39; 95% CI: 0.34–6.44) (*p* = 0.019), whereas no significant differences were observed at other time points. Analgesic use was reported by 12.9% of patients in the TeM group and by none in the SRN group (*p* = 0.004). Responder analysis confirmed that the SRN system resulted in a higher proportion of pain-free patients on day 3 (RR = 0.83; 95% CI: 0.70–0.98; *p* = 0.027). Both reciprocating and continuous rotary systems showed a gradual reduction in postoperative pain after single-visit endodontic treatment. A modest and short-lived difference was observed on day 3, with slightly higher pain levels in the reciprocating group, suggesting a temporary variation in the early postoperative response rather than a clinically decisive effect of instrument kinematics.

**Supplementary Information:**

The online version contains supplementary material available at https://doi.org/10.1007/s10266-026-01316-4.

## Introduction

Root canal treatment aims to eliminate infected or necrotic pulp tissue, disinfect, and shape the root canal system, and obturate it hermetically to preserve long-term tooth function and periapical health [1]. Despite its high success rate, root canal treatment is an invasive procedure that inevitably involves mechanical and chemical irritation of periapical tissues during canal instrumentation and irrigation. These factors may provoke an inflammatory response in the periradicular area, leading to postoperative discomfort or pain.

Postoperative endodontic pain is primarily attributed to periapical inflammation caused by mechanical, chemical, or microbial irritants extruded beyond the apical foramen during treatment [2]. Factors that may influence the occurrence of postoperative pain include the clinician’s experience level, the patient’s gender and systemic health condition, the clinical status of the pulpal and periapical tissues, the presence and severity of preoperative pain, the characteristics of the file system used during canal preparation, apical patency control, irrigation protocol, and obturation technique [3]. While bacterial species and their pathogenic potential cannot be controlled, chemomechanical disinfection protocols and the instruments used are operator-dependent variables [4]. Among these, the characteristics and kinematics of the file system play a key role as the severity of periapical inflammation is closely related to the amount of apically extruded debris during chemomechanical preparation [5].

The kinematic motion of nickel–titanium (NiTi) instruments may influence debris extrusion by altering cutting dynamics, file engagement, and canal shaping behavior [5, 6]. Experimental studies have shown inconsistent results regarding debris extrusion associated with reciprocating versus continuous rotary motion, with some reporting greater extrusion with reciprocation [7, 8] and others favoring continuous rotation [9, 10]. These discrepancies may be partly explained by differences in file design and metallurgical properties, which affect cutting efficiency and debris transportation [5, 6, 11]. From a biological perspective, apically extruded debris can stimulate the release of inflammatory mediators, such as prostaglandins, histamine, and bradykinin, thereby amplifying periapical inflammation and pain perception [12–14].

Despite the clinical relevance of postoperative pain as a patient-centered outcome, well-controlled clinical trials investigating the effect of file kinematics on postoperative pain remain limited, particularly in vital, single-rooted teeth treated under standardized irrigation and obturation protocols [14–17]. This limitation reduces the ability to isolate the specific influence of instrument kinematics from other confounding variables.

In this context, the T-endo MUST (TeM) system (Dentac, Istanbul, Turkey), which operates with reciprocating motion and TM-Wire heat treatment [18], and the Scope RS Narrow (SRN) system (Scope, Yozgat, Turkey), which features Gold heat treatment and continuous rotation [19], represent two contemporary NiTi systems with distinct kinematic and metallurgical characteristics [20, 21]. These differences may lead to different periapical tissue responses and postoperative pain profiles.

Therefore, the aim of this prospective clinical study was to compare postoperative pain intensity and analgesic consumption following single-visit root canal treatment of vital mandibular premolars performed with reciprocating (TeM) and continuous rotary (SRN) file systems. This study tested the hypothesis that rotary and reciprocating kinematics would result in similar postoperative pain levels at day 3 after treatment, which was defined as the primary endpoint. Other days (1,5, and 7) and analgesic consumption were defined as the secondary outcomes.

## Materials and methods

This study was conducted and reported in accordance with the TREND statement, and the completed TREND checklist is provided as Supplementary File 1. This prospective clinical study was approved by the Non-Interventional Clinical Research Ethics Committee of Recep Tayyip Erdoğan University (Approval No: 2025/100; Date: 20.03.2025) and conducted at the Department of Endodontics, Faculty of Dentistry, Recep Tayyip Erdoğan University.

### Sample size calculation

The sample size was determined using the G*Power software (version 3.1.9.7) to ensure adequate statistical power for the study. The assumed effect size was derived from the difference in postoperative pain scores on postoperative day 1 reported by Kurnaz [22] in a randomized clinical trial comparing reciprocating and continuous rotary instrumentation systems. Based on these data, the effect size was calculated as Cohen’s d = 0.534, corresponding to a medium effect size. Using an a priori power of 80% and a two-sided significance level of 0.05, the required total sample size was calculated as 116 participants, with a minimum of 58 participants per group.

### Inclusion and exclusion criteria

Participants were required to be ≥ 18 years old, systemically healthy, mentally competent, cooperative during treatment, and willing to provide written informed consent. Only vital mandibular premolar teeth with complete root formation and suitable for single-visit treatment were included. Exclusion criteria comprised systemic disease, current use of antibiotics or corticosteroids, preoperative analgesic use, pregnancy or lactation, teeth with incomplete root formation, resorption, advanced periodontal bone loss, pathological mobility, root fracture, periapical cysts, or requiring retreatment.

All participants received detailed verbal and written information about the study objectives, procedures, and potential risks. Informed consent was obtained in accordance with ethical standards. Sociodemographic data, clinical findings, diagnosis, treatment details, and analgesic use were recorded on a standardized case report form (Supplementary File 2).

### Application of the visual analog scale

**Fig. 1:**
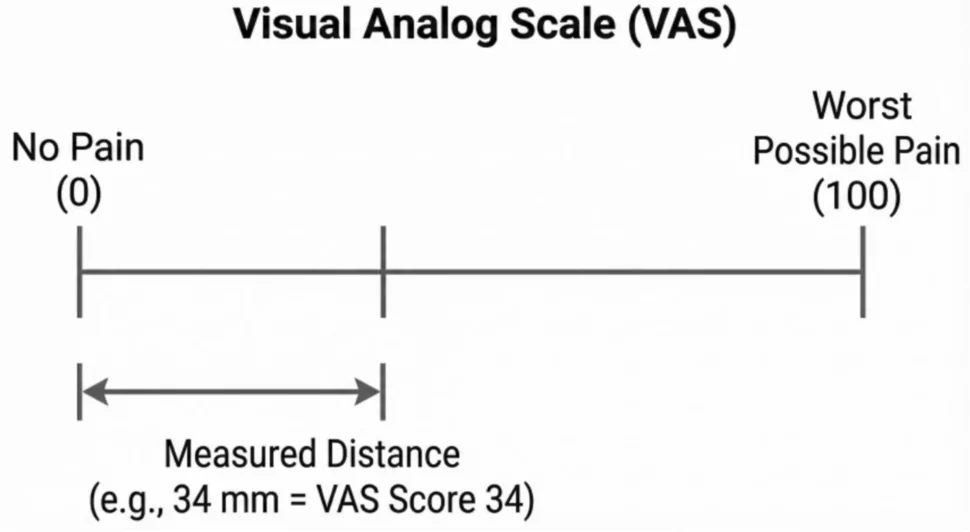
Schematic presentation of the 100-mm Visual Analog Scale (VAS) used in the study for pain intensity assessment and the scoring method

Before treatment, all patients were instructed on how to use the Visual Analog Scale (VAS) to assess pain intensity, as illustrated in Fig. 1. The VAS consisted of a 100-mm horizontal line anchored by the verbal descriptors 'No pain' (0) and 'Worst possible pain' (100). Patients were asked to mark a vertical line on the scale at the point corresponding to their pain level. The pain score was then determined by measuring the distance in millimeters from the left end (0) to the patient's mark [23]. Each participant recorded their baseline pain level prior to treatment using this method. To monitor postoperative pain, each patient received a standardized pain diary and was asked to record the highest pain intensity experienced during the first 24 h, between 48 and 72 h, on the fifth day, and on the seventh day. Patients also documented any analgesic medication taken, including type, dosage, and timing. During follow-up visits, the completed pain diaries were collected by the investigators. Participants who failed to attend or submitted incomplete forms were contacted by telephone to obtain missing data, ensuring complete documentation for analysis.

### Endodontic treatment protocol

To minimize selection bias between the two systems, eligible cases presenting consecutively to the clinic were assigned alternately to either the TeM or SRN rotary system. This alternate assignment of consecutive cases was used to ensure balanced group sizes; however, true randomization and allocation concealment were not performed.

All clinical procedures were performed in a single visit by an experienced operator to ensure standardization. After administration of local anesthesia, rubber dam isolation was achieved, an access cavity was prepared, and the working length was determined using an electronic apex locator and confirmed radiographically.

In all cases, irrigation was performed using 2.5% sodium hypochlorite (NaOCl) and 17% ethylenediaminetetraacetic acid (EDTA). A total volume of 10 mL of 2.5% NaOCl and 5 mL of 17% EDTA was used per canal. Irrigation was delivered using a side-vented needle inserted 1 mm short of the working length, and no irrigant activation system was employed. Canal preparation in both groups was completed to a final apical size of 25/0.04. After drying the canals, obturation was completed using appropriate gutta-percha cones and MTA CEM sealer with the single-cone technique, followed by placement of the definitive restoration.

Routine occlusal reduction was not performed; however, after completion of root canal treatment, occlusal contacts were carefully checked to ensure proper alignment and to confirm that no high points remained. Analgesic intake was not standardized; no postoperative analgesic regimen was prescribed, and patients used analgesics only if they personally felt the need. All treatments were performed by a single experienced endodontist with more than 10 years of clinical practice.

### Differences between groups

In both groups, canal preparation was performed using a torque-controlled endodontic motor (Ai; Woodpecker, Guilin, China) operating at 300 revolutions per minute (rpm). Both systems were used according to the manufacturers’ instructions. The torque settings, rotational speed, and file sequence for both systems are summarized in Table 1.

- Group 1 (SRN System): Preparation was performed at a torque of 1.5–2 N·cm in continuous rotation. The following sequence of files was used: N1 Opener (20/0.08), N2 PathFile (17/0.03), N3 Finisher (20/0.04), and N4 Finisher (25/0.04).
- Group 2 (TeM System): Preparation was performed at a torque of 4 N·cm in reciprocating motion. TG (13/0.04) and M25 files were used sequentially. The motor was set to 160° counterclockwise and 40° clockwise rotation angles. Files were advanced with 2-mm pecking motions; whenever increased resistance was felt, the file was withdrawn, cleaned, and the canal was irrigated with 2 mL of 2.5% NaOCl. Apical patency was maintained using a #10 K-file.

**Table 1:** Torque settings, rotational speed, and file sequence for both systems

Parameter	SRN system (Rotational)	TeM system (Reciprocating)
Kinematics	Continuous rotation	Reciprocating motion
Torque	1.5–2 N·cm	4 N·cm
Rotational speed	300 rpm	300 rpm (160° CCW / 40° CW)
File sequence	N1 → N2 → N3 → N4	TG → M25
Number of files	4	2

### Statistical analysis

All analyses were performed using the Jamovi statistical software (version 2.3.28). Demographic variables were compared between rotary file systems using the chi-square test for categorical data and the Student’s t test for normally distributed continuous data. Pain scores did not follow a normal distribution; therefore, nonparametric tests were applied. Mann–Whitney U test was used for intergroup comparisons, and changes in pain over time were evaluated with the Friedman test. Additionally, a responder analysis was conducted to determine the proportion of patients achieving clinically meaningful pain reduction, defined as ≥ 30% or ≥ 50% decrease from baseline or a VAS score < 10. Risk ratios (RRs) were estimated using modified Poisson regression models adjusted for baseline pain, age, gender, and treated tooth. A *p* value < 0.05 was considered statistically significant.

## Results

**Fig. 2:**
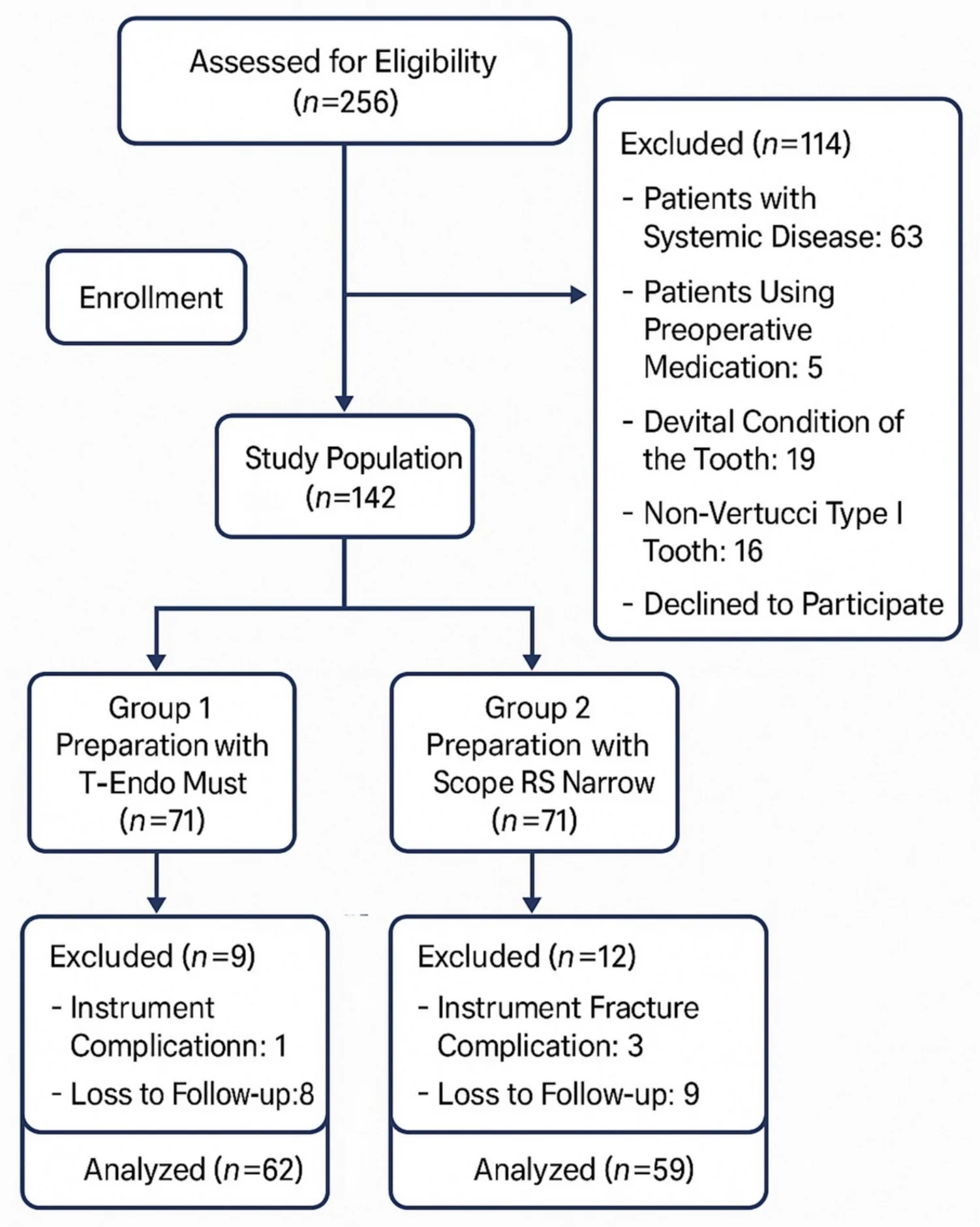
Flow diagram of the study

A total of 256 patients who presented to the Department of Endodontics, Faculty of Dentistry, Recep Tayyip Erdogan University, for root canal treatment were assessed for eligibility. After evaluation, 114 patients were excluded due to systemic disease (*n* = 63), preoperative medication use (*n* = 5), non-vital teeth (*n* = 19), canal morphology other than Vertucci Type I (*n* = 16), or refusal to participate (*n* = 11). The remaining 142 patients met the inclusion criteria and were randomly allocated into two equal groups according to the rotary file system used: the TeM system (*n* = 71) and the SRN system (*n* = 71). During follow-up, one patient in the TeM group was excluded due to instrument fracture, and eight were lost to follow-up. In the SRN group, three patients experienced instrument fracture, and nine were lost to follow-up. Consequently, 121 patients were included in the final analysis: 62 in the TeM group and 59 in the SRN group. Patient screening, allocation, exclusions, and follow-up are summarized in a flow diagram (Fig. 2).

The demographic and clinical characteristics of participants are presented in Table 2. Both groups were comparable in terms of gender, age, marital status, education level, and distribution of treated teeth (*p* > 0.05 for all). The mean age was 38.0 ± 12.0 years in the TeM group and 37.1 ± 12.2 years in the SRN group. The majority of participants in both groups were married (69.4% and 67.8%, respectively) and had completed high school education (40.3% and 42.4%, respectively). Teeth #45 and #35 were the most frequently treated in the TeM and SRN groups, respectively. The mean procedure duration was similar between groups (50.6 ± 13.9 min vs. 52.8 ± 15.8 min; *p* = 0.425). However, analgesic use was significantly higher in the TeM group (12.9%) than in the SRN group (0%) (*p* = 0.004).

**Table 2:** Distribution of Demographic Characteristics According to Two Different Rotary Instrument Systems

	T-endo must (*n* = 62)	Scope RS Narrow (*n* = 59)	Total (*n* = 121)	*p* value
Gender				0.904^1^
Male	28 (45.2%)	26 (44.1%)	54 (44.6%)	
Female	34 (54.8%)	33 (55.9%)	67 (55.4%)	
Age				0.694^2^
Mean ± SD	38.0 ± 12.0	37.1 ± 12.2	37.5 ± 12.0	
Min–Max	18–59	18–64	18–64	
Marital Status				0.854^1^
Married	43 (69.4%)	40 (67.8%)	83 (68.6%)	
Single	19 (30.6%)	19 (32.2%)	38 (31.4%)	
Education				0.345^1^
Primary Education	23 (37.1%)	16 (27.1%)	39 (32.2%)	
High School	25 (40.3%)	25 (42.4%)	50 (41.3%)	
University	14 (22.6%)	16 (27.1%)	30 (24.8%)	
Illiterate	0 (0.0%)	2 (3.4%)	2 (1.7%)	
Tooth Number				0.464^1^
34	8 (12.9%)	7 (11.9%)	15 (12.4%)	
35	21 (33.9%)	23 (39.0%)	44 (36.4%)	
44	5 (8.1%)	9 (15.3%)	14 (11.6%)	
45	28 (45.2%)	20 (33.9%)	48 (39.7%)	
Procedure Duration				0.425^2^
Mean ± SD	50.6 ± 13.9	52.8 ± 15.8	51.7 ± 14.8	
Min–Max	25–85	20–90	20–90	
Analgesic Usage				**0.004**^**1**^
Yes	8 (12.9%)	0 (0.0%)	8 (6.6%)	
No	54 (87.1%)	59 (100.0%)	113 (93.4%)	

n (%), ^1^ Chi-squared test, ^2^ Students t test

When postoperative pain was evaluated according to gender, no statistically significant differences were observed between male and female participants at any time interval (*p* > 0.05). Similarly, age did not have a significant effect on postoperative pain, as participants aged below and above 35 years reported comparable pain levels across all time points (*p* > 0.05) (Table 3).

**Table 3:** Comparison of Postoperative Pain Scores by Gender and Age

Time Point	Group	Mean ± SD	95% CI	Median (Min–Max)	p value^1^
Preoperative	Female	14.85 ± 27.37	8.17 to 21.53	0 (0–100)	0.753
	Male	13.70 ± 22.53	7.55 to 19.85	0 (0–80)	
	≤ 35 yrs	16.92 ± 27.91	9.71 to 24.13	0 (0–100)	0.394
	> 35 yrs	11.80 ± 22.23	6.11 to 17.49	0 (0–100)	
Day 1	Female	13.09 ± 22.09	7.70 to 18.48	0 (0–80)	0.243
	Male	9.35 ± 20.49	3.76 to 14.94	0 (0–80)	
	≤ 35 yrs	10.53 ± 20.13	5.33 to 15.73	0 (0–70)	0.615
	> 35 yrs	12.30 ± 22.69	6.49 to 18.11	0 (0–80)	
Day 3	Female	6.64 ± 15.89	2.76 to 10.52	0 (0–80)	0.335
	Male	5.00 ± 13.97	1.19 to 8.81	0 (0–60)	
	≤ 35 yrs	5.58 ± 14.41	1.86 to 9.30	0 (0–60)	0.871
	> 35 yrs	6.23 ± 15.72	2.20 to 10.26	0 (0–80)	
Day 5	Female	4.25 ± 14.68	0.67 to 7.83	0 (0–80)	0.142
	Male	1.20 ± 6.93	-0.69 to 3.09	0 (0–50)	
	≤ 35 yrs	1.33 ± 6.76	-0.42 to 3.08	0 (0–50)	0.228
	> 35 yrs	4.43 ± 15.31	0.51 to 8.35	0 (0–80)	
Day 7	Female	1.87 ± 10.18	-0.61 to 4.35	0 (0–80)	0.168
	Male	0.93 ± 6.80	-0.93 to 2.79	0 (0–50)	
	≤ 35 yrs	0.50 ± 2.72	-0.20 to 1.20	0 (0–20)	0.984
	> 35 yrs	2.38 ± 12.10	-0.72 to 5.48	0 (0–80)	

^1^ Mann–Whitney U test

**Fig. 3:**
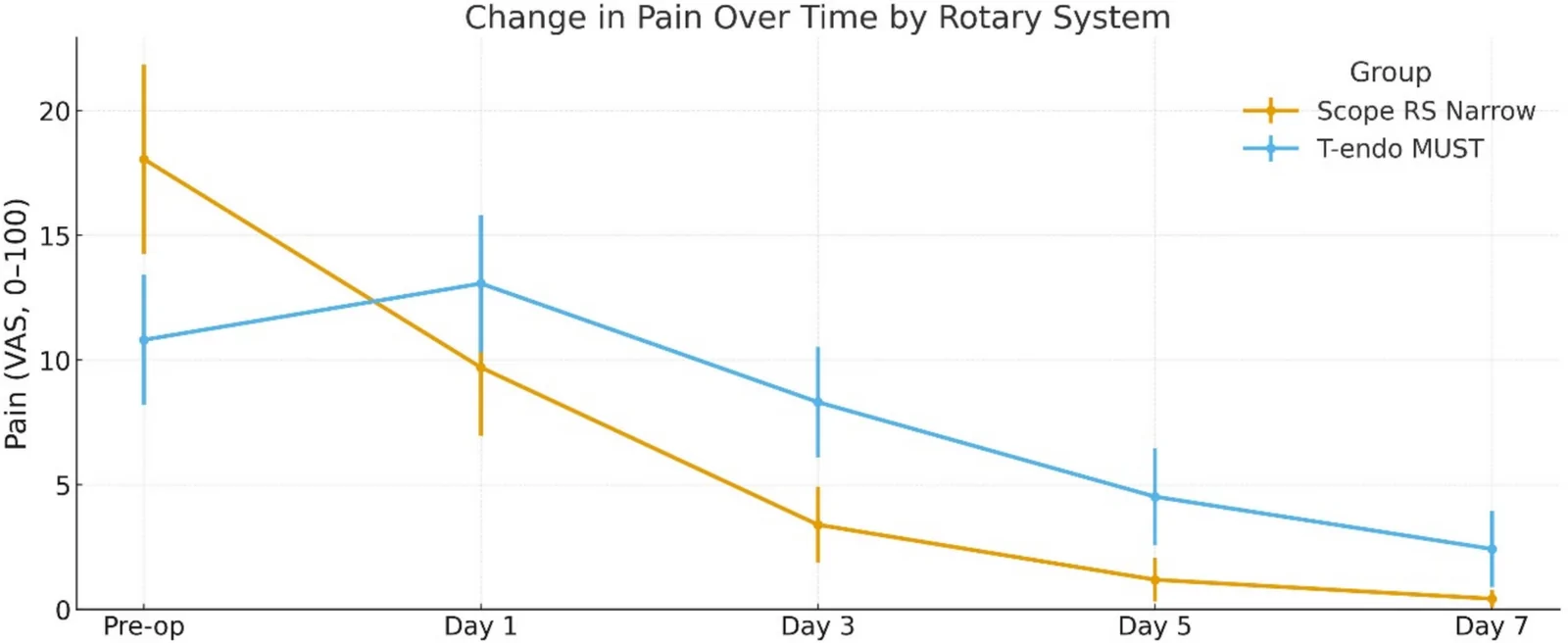
Time course of postoperative pain by rotary system (mean ± SE)

When pain intensity was compared between the two rotary file systems, the TeM group demonstrated significantly higher postoperative pain (8.31; 95% CI: 3.89–12.72) on the third day compared with the SRN group (3.39; 95% CI: 0.34–6.44) (*p* = 0.019). At other time points, no statistically significant differences were observed (*p* > 0.05) (Table 4, Fig. 3).

**Table 4:** Comparison of Postoperative Pain by Rotary Instrument Systems

Time point	System	Mean (95% CI)	SD	Median (Min–Max)	*p* value
Preoperative	T-endo must	10.81 (5.60–16.01)	20.51	0 (0–70)	0.138^1^
	Scope	18.05 (10.46–25.64)	29.11	0 (0–100)	
Postoperative Day 1	T-endo must	13.06 (7.56–18.57)	21.68	0 (0–80)	0.090^1^
	Scope	9.70 (4.19–15.20)	21.12	0 (0–80)	
Postoperative Day 3	T-endo must	8.31 (3.89–12.72)	17.39	0 (0–80)	**0.019**^1^
	Scope	3.39 (0.34–6.44)	11.69	0 (0–50)	
Postoperative Day 5	T-endo must	4.52 (0.66–8.38)	15.20	0 (0–80)	0.084^1^
	Scope	1.19 (0.00–2.94)	6.72	0 (0–50)	
Postoperative Day 7	T-endo must	2.42 (0.00–5.47)	12.00	0 (0–80)	0.432^1^
	Scope	0.42 (0.00–1.12)	2.67	0 (0–20)	

Values are presented as mean VAS score (95% confidence interval), standard deviation (SD), and median (minimum–maximum). ^1^ Mann–Whitney *U* test

**Fig. 4:**
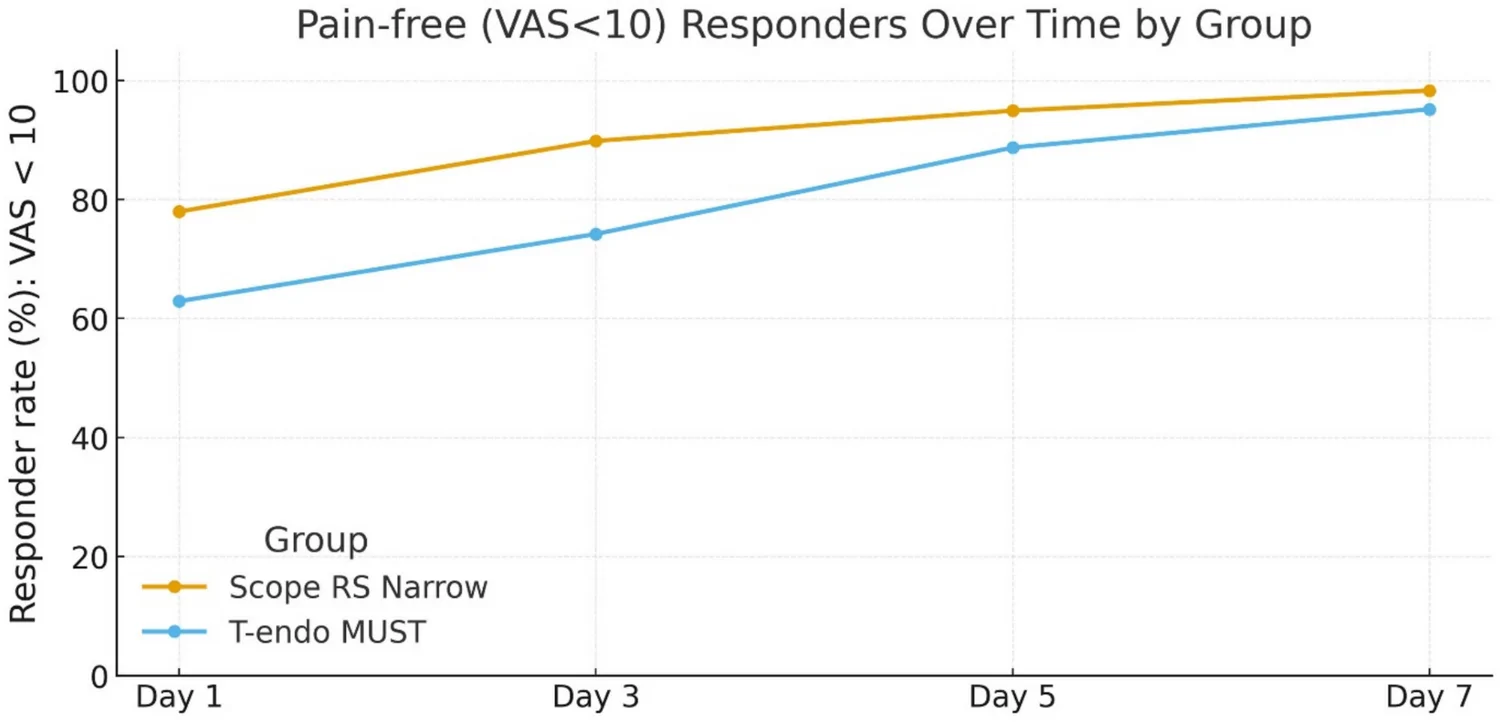
Proportion of Pain-Free Responders (VAS < 10) Over Time by Rotary File System

In the responder analysis, none of the evaluated definitions of clinical improvement— ≥ 30% or ≥ 50% reduction in pain or achieving a VAS score below 10—showed consistent significant differences between the rotary systems across the follow-up period. While both groups demonstrated a steady increase in the proportion of patients achieving meaningful pain relief over time, a statistically significant difference was observed only on the third postoperative day for the VAS < 10 criterion (RR = 0.83; 95% CI 0.70–0.98; *p* = 0.027), indicating that fewer patients in the TeM group were completely pain-free at this point compared with the SRN group (Fig. 4). By Day 7, nearly all patients in both groups reached minimal or no pain levels, and intergroup differences were no longer evident. These findings suggest that, although the overall clinical course of postoperative pain reduction was comparable between systems, SRN provided slightly faster short-term pain relief (Table 5).

**Table 5:** Responder (MCID) Analysis — Adjusted Risk Ratios for Pain Reduction and Relief (TeM vs SRN)

Outcome (Responder Definition)	Time Point	Events (n)	Adjusted RR (95% CI)	*p* value
≥ 30% reduction	Day 1	31	1.00 (0.56 – 1.78)	0.996
≥ 30% reduction	Day 3	36	0.75 (0.46 – 1.21)	0.231
≥ 30% reduction	Day 5	38	0.89 (0.57 – 1.40)	0.609
≥ 30% reduction	Day 7	39	0.96 (0.61 – 1.50)	0.847
≥ 50% reduction	Day 1	28	0.86 (0.48 – 1.56)	0.620
≥ 50% reduction	Day 3	35	0.78 (0.47 – 1.30)	0.338
≥ 50% reduction	Day 5	37	0.82 (0.52 – 1.30)	0.401
≥ 50% reduction	Day 7	39	0.96 (0.61 – 1.50)	0.847
VAS < 10 (pain-free)	Day 1	85	0.80 (0.64 – 1.01)	0.062
VAS < 10 (pain-free)	Day 3	99	0.83 (0.70 – 0.98)	0.027 *
VAS < 10 (pain-free)	Day 5	111	0.94 (0.84 – 1.04)	0.209
VAS < 10 (pain-free)	Day 7	117	0.97 (0.91 – 1.04)	0.371

^*^p < 0.05 (statistically significant)

RR < 1 indicates lower likelihood of achieving the response in the T-endo MUST group relative to Scope RS Narrow

## Discussion

This study aimed to compare postoperative pain following canal preparation performed with reciprocating (TeM) and continuous rotary (SRN) systems. The null hypothesis that there would be no difference between the two systems at any time point was rejected, as a significant difference was found on the third postoperative day, while no significant differences were observed at 24 h, or on the fifth and seventh days. Although numerous studies have investigated postoperative pain after root canal treatment using various NiTi rotary file systems [24–26], no previous clinical study has directly compared SRN and TeM. Thus, to the best of our knowledge, this research represents the first clinical investigation to directly evaluate the influence of these two systems on postoperative pain profiles following endodontic treatment.

The results of the present study indicate that postoperative endodontic pain is largely governed by a neurophysiological mechanism independent of gender and age. Inflammation of pulpal and periapical tissues activates nociceptive signaling in primary afferent neurons, causing transient pain typically confined to a localized inflammatory response without gender-based differences [27]. Although some studies have reported that females may have a lower pain threshold, such differences are more commonly observed in chronic pain models rather than in short-term, controlled inflammatory conditions such as postoperative endodontic pain [28, 29]. Similarly, the mean age was 38.0 ± 12.0 years in the TeM group and 37.1 ± 12.2 years in the SRN group, with no statistically significant relationship between age and postoperative pain intensity at any time point. This finding is consistent with previous studies suggesting that postoperative pain is influenced primarily by local inflammatory responses and instrumentation technique rather than demographic variables [30, 31].

One of the main causes of postoperative pain is the extrusion of dentin debris, pulpal remnants, and irrigating solutions into periapical tissues during chemomechanical preparation [6, 32, 33]. Such extrusion triggers an acute inflammatory reaction by stimulating the release of mediators, such as prostaglandins, histamine, and bradykinin. Previous studies have reported that reciprocating systems, due to their more aggressive cutting motion, may result in greater apical debris extrusion, thereby enhancing the inflammatory response and increasing postoperative pain [34]. In contrast, continuous rotary systems facilitate smoother debris removal along the canal path, which may reduce periapical irritation and, consequently, pain severity [35]. However, other studies have shown that single-file reciprocating systems may extrude less debris apically than multi-file rotary systems [34]. Similarly, Koçak et al. [6] found no significant difference in apical debris extrusion between rotary and reciprocating systems, suggesting that factors, such as file design, taper, and cross-sectional geometry also play substantial roles.

Recent clinical studies published between 2022 and 2025 have further explored the relationship between file motion and postoperative pain, providing additional context for the present findings. A randomized clinical trial comparing ProTaper Next® and Reciproc® instruments reported no statistically significant difference in postoperative pain intensity up to one week after root canal preparation, suggesting comparable outcomes between the two kinematic types [36]. Similarly, other randomized clinical trials evaluating rotary and reciprocating single-file systems at 24, 48, and 72 h have also found no meaningful difference in postoperative pain severity [37]. In addition, an ongoing randomized clinical trial comparing the ProTaper Ultimate and Reciproc Blue systems highlights continued scientific interest in the impact of kinematics on patient-centered outcomes [38]. Complementing these clinical findings, an umbrella review and meta-analysis reported that while rotary systems may be associated with slightly lower postoperative pain levels than reciprocating systems, such differences are likely influenced by multiple factors, including metallurgy, taper, and apical debris extrusion, rather than motion type alone [39].

In the present study, the highest postoperative pain scores were recorded within the first 24 h, consistent with the literature reporting that pain following root canal treatment is most intense during this period [36, 40]. This phenomenon can be attributed to the acute inflammatory reaction that develops in the periapical tissues after instrumentation. During this phase, proinflammatory mediators, such as histamine, prostaglandins, bradykinin, TNF-α, and IL-1β, are released, sensitizing peripheral nociceptors and lowering the pain threshold, which leads to intense pain [41, 42]. Although the acute phase continues on the second day, the production of these mediators begins to decline as macrophage activity increases, initiating the healing process [43]. By the third day, anti-inflammatory cytokines and resolvins begin to counteract nociceptor sensitization, resulting in progressive pain reduction, which becomes prominent after the fifth day [41, 42].

In both groups, the highest pain scores were recorded within the first 24 h, with no statistically significant differences between the TeM and SRN systems at that point. However, on the third day, the TeM group exhibited significantly higher pain levels compared with the SRN group. This finding can be explained by greater apical debris extrusion associated with the reciprocating motion. Fundaoğlu Küçükekenci [8] similarly reported higher debris extrusion with the TeM system, supporting our interpretation that this mechanical difference has a clinical impact. After the third day, as inflammation entered the resolution phase, the activity of anti-inflammatory mediators increased, tissue edema subsided, and pain progressively decreased [41, 42]. Consistent with other studies [24–26, 40], the early higher pain levels observed in the TeM group diminished over time, and by the fifth and seventh days, no significant differences remained between groups. This pattern reflects the natural resolution dynamics of acute inflammation.

Although a statistically significant difference in postoperative pain was observed between the groups on postoperative day 3, the magnitude of this difference was modest and transient. The mean intergroup difference was approximately 5 mm on a 100-mm VAS, which is below commonly reported thresholds for a minimal clinically important difference in acute postoperative pain [44, 45]. Moreover, no significant differences were detected at other postoperative time points, indicating that the observed effect was limited to the early healing phase and resolved within a short period. From a clinical perspective, these findings suggest that while file kinematics may influence short-term postoperative discomfort, the impact on overall patient experience and clinical decision-making is likely limited.

The overall analgesic consumption rate in the present study was 12.9%, observed exclusively in the TeM group, whereas no patients in the SRN group required analgesics. This result lies at the lower end of the wide range reported in the literature (7.7%–89.6%) [46, 47]. While some studies have reported higher analgesic use with reciprocating systems (15), others found no significant difference [36, 40]. These inconsistencies may reflect methodological variations and differences in study populations. The complete absence of analgesic use in the SRN group underscores the potential of continuous rotation to reduce postoperative discomfort without pharmacological intervention. Although this difference was limited to a short-term period, it may lead to improved patient satisfaction, especially in outpatient care. The current findings support the notion that reciprocating kinematics may contribute to increased apical debris extrusion, leading to greater inflammatory response and pain [8].

The fact that this significant difference was only seen on the third day, with no differences at 24 h or beyond day five, aligns with the natural course of acute inflammation. These findings emphasize that analgesic requirement should be viewed not only as an indicator of procedural efficacy but also of patient comfort. Postoperative pain is subjective and influenced by individual factors, such as pain threshold and previous experience. To reduce bias, patients were asked to record their maximum pain level prior to taking medication and to document analgesic use. This ensured consistent reporting of peak pain and improved the quality of the data. From a clinical standpoint, SRN was associated with slightly lower pain and no reported analgesic use; however, the observed difference was modest and limited to Day 3. Therefore, these findings should be interpreted as reflecting a small short-term effect rather than a basis for recommending one kinematic approach over another.

The primary limitation of this study was that a double-blind design could not be implemented, as the operator could not be blinded to the instrumentation system used. In addition, patient allocation was performed using an alternate assignment of consecutively admitted cases without allocation concealment, which should be acknowledged as a methodological limitation, although this approach was intended to ensure a balanced distribution between groups. The absence of operator blinding and a standardized postoperative analgesic protocol, with analgesic intake recorded but not protocolized, may have introduced additional variability in pain reporting. Furthermore, the single-center design and relatively limited sample size, together with the restriction of the study population to vital, single-rooted mandibular premolars, may limit the external validity and generalizability of the findings to other tooth types or clinical scenarios. Subjective variability in individual pain thresholds may also have contributed to random error in VAS assessments, and psychological or cultural factors influencing pain perception were not controlled. Nonetheless, the high level of methodological standardization achieved including uniform treatment protocols, a single experienced operator, and controlled clinical conditions is likely to have mitigated these limitations. Future multicenter studies with larger and more diverse samples, as well as investigations incorporating biochemical markers of inflammation, are warranted to further clarify the clinical relevance and underlying mechanisms of postoperative pain.

## Conclusion

Within the limitations of this single-center prospective clinical study, both reciprocating (TeM) and continuous rotary (SRN) file systems demonstrated a gradual reduction in postoperative pain following single-visit root canal treatment. A transient difference was observed on the third postoperative day, with slightly higher pain scores and limited analgesic use in the TeM group; however, this difference was modest in magnitude and resolved within a short period. These findings suggest a temporary variation in early postoperative response rather than a sustained or clinically decisive effect attributable to kinematic motion.

From a clinical perspective, however, the observed between-group difference was modest and short-lived, and therefore may not be sufficient to justify the selection of an instrumentation system based solely on kinematic motion. Future multicenter trials with larger and more diverse populations, along with investigations into biochemical markers of inflammation, are warranted to confirm these results and clarify the biological mechanisms underlying postoperative pain.

## Supplementary Information

Below is the link to the electronic supplementary material.

Supplementary file1 (PDF 556 KB)

Supplementary file2 (DOCX 18 KB)

## Data Availability

The datasets generated and/or analyzed during the current study are not publicly available considering that we have not required consents to publish this data, but are available from the corresponding author on reasonable request.
