# Supplementary material for: Prospective comparative study on postoperative pain after single-visit root canal treatment using rotary and reciprocating kinematics
Source: Odontology. 2026 Jan 27;114(4):2493–503. doi: 10.1007/s10266-026-01316-4 (PMC13631025; doi:10.1007/s10266-026-01316-4)
Supplement: Supplementary file 2 [file 10266_2026_1316_MOESM2_ESM.docx]

| Supplementary File 2. Example of the Study Form |  |
| --- | --- |
| Date of treatment: |  |
| Patient Name: |  |
| Patient’s Age: |  |
| Patient’s Gender: | - Male - Female |
| Marital Status: |  |
| Educational Level: | - Primary Education - High School - University Education - Illiterate |
| Medications Used by the Patient: |  |
| Tooth Number: |  |
| Tooth Diagnosis: |  |
| Duration of the Procedure: |  |
| Was Anesthesia Administered During the Procedure? If Yes, Specify the Number of Cartridges: | - No - Yes 1 - Yes 2 - Yes 3 |
| Type of Local Anesthetic Used: |  |
| Endodontic File System Used: | - T-endo MUST - Scope RS Narrow |
| The Final Rotary File Used During the Procedure: | - 25/06 - 25/04 |
| Was an Analgesic Taken After the Treatment? | - No - Yes, within the first 24 hours - Yes, for 3 days - Yes, for 5 days - Yes, for 7 days |
| Which Analgesic Was Used? |  |
| Pain Perceived Before the Treatment (0–100 Scale): |  |
| Maximum Pain Intensity on Day 1 (0–24 Hours) (0–100 Scale): |  |
| Maximum Pain Intensity Experienced on Day 3 After Treatment (48–72 Hours) (0–100 Scale): |  |
| Maximum Pain Intensity Experienced on Day 5 After Treatment (0–100 Scale): |  |
| Maximum Pain Intensity Experienced on Day 7 After Completion of Treatment (0–100 Scale): |  |
